# Morphostasis in a novel eukaryote illuminates the evolutionary transition from phagotrophy to phototrophy: description of *Rapaza viridis *n. gen. et sp. (Euglenozoa, Euglenida)

**DOI:** 10.1186/1471-2148-12-29

**Published:** 2012-03-08

**Authors:** Aika Yamaguchi, Naoji Yubuki, Brian S Leander

**Affiliations:** 1 The Department of Botany and Zoology, Beaty Biodiversity Research Center and Museum, University of British Columbia, 6270 University Blvd., Vancouver, British ColumbiaV6T 1Z4, Canada

## Abstract

**Background:**

Morphostasis of traits in different species is necessary for reconstructing the evolutionary history of complex characters. Studies that place these species into a molecular phylogenetic context test hypotheses about the transitional stages that link divergent character states. For instance, the transition from a phagotrophic mode of nutrition to a phototrophic lifestyle has occurred several times independently across the tree of eukaryotes; one of these events took place within the Euglenida, a large group of flagellates with diverse modes of nutrition. Phototrophic euglenids form a clade that is nested within lineages of phagotrophic euglenids and that originated through a secondary endosymbiosis with green algae. Although it is clear that phototrophic euglenids evolved from phagotrophic ancestors, the morphological disparity between species representing these different nutritional modes remains substantial.

**Results:**

We cultivated a novel marine euglenid, *Rapaza viridis *n. gen. et sp. ("green grasper"), and a green alga, *Tetraselmis *sp., from the same environment. Cells of *R. viridis *were comprehensively characterized with light microscopy, SEM, TEM, and molecular phylogenetic analysis of small subunit rDNA sequences. Ultrastructural and behavioral observations demonstrated that this isolate habitually consumes a specific strain of *Tetraselmis *prey cells and possesses a functional chloroplast that is homologous with other phototrophic euglenids. A novel feeding apparatus consisting of a reduced rod of microtubules facilitated this first and only example of mixotrophy among euglenids. *R. viridis *also possessed a robust photoreception apparatus, two flagella of unequal length, euglenoid movement, and a pellicle consisting of 16 strips and one (square-shaped) whorl of posterior strip reduction. The molecular phylogenetic data demonstrated that *R. viridis *branches as the nearest sister lineage to phototrophic euglenids.

**Conclusions:**

The unusual combination of features in *R. viridis *combined with its molecular phylogenetic position completely conforms to the expected transitional stage that occurred during the early evolution of phototrophic euglenids from phagotrophic ancestors. The marine mixotrophic mode of nutrition, the preference for green algal prey cells, the structure of the feeding apparatus, and the organization of the pellicle are outstanding examples of morphostasis that clarify pivotal stages in the evolutionary history of this diverse group of microbial eukaryotes.

## Background

### Morphostasis and transitional traits

The reconstruction of early stages in the evolution of complex characters requires awareness of different degrees of morphostasis in character states as reflected in the unity and diversity of organisms. Evolutionary biologists who study groups of organisms with rich fossil records (e.g., molluscs, vertebrates, diatoms and foraminiferans) have the luxury of comparing both extinct and extant species to infer patterns of character evolution. Evolutionary biologists focused on microbial eukaryotes are most often restricted to comparisons of character states present in extant species to infer patterns of character evolution [1]. As expected, many species have retained traits that are completely compatible with those that are either inferred or observed to have been present in distant ancestors (e.g., *Nautilus, Latimeria, Acanthostega, Diarthrognathus *and *Archaeopteryx*; [2-4]). The discovery, characterization, and molecular phylogenetic analyses of species like these test hypotheses about trait evolution and illuminate the transitional stages that link divergent character states.

Among the most significant events in the evolutionary history of eukaryotes is the transition from a phagotrophic mode of life to a phototrophic mode of life, which has occurred several times independently [5-7]. This switch in nutritional mode has profound effects on the overall morphology and behavior of the descendant species. For instance, phagotrophic organisms tend to be highly motile in order to acquire food items and are usually composed of pliable cells or tissues that can accommodate ingested material; phototrophic organisms, by contrast, tend to be non-motile, sessile or planktonic and are usually composed of cells or tissues encased in a rigid wall for protection or structural integrity. The evolutionary transition from phagotrophy to phototrophy is therefore non-trivial, and the intermediate stages that bridge the morphological traits associated with these two lifestyles are important to understand. With this in mind, we have discovered and characterized a novel marine species with a suite of character states that completely conforms to the inferred transitional stage between the phagotrophic and phototrophic lifestyles present in a diverse group of microbial eukaryotes, namely the Euglenida.

### A brief overview of euglenid biology and evolutionary history

The Euglenida is a large group of marine and freshwater flagellates that contains more than 1,000 described species with diverse modes of nutrition, including phagotrophy (bacterivory and eukaryovory), osmotrophy (sensu stricto) and phototrophy [8]. The best synapomorphy for this group of microbial eukaryotes is a cell surface consisting of longitudinally or helically arranged pellicle strips that articulate along their lateral margins [9]. Many species of phagotrophic, osmotrophic (sensu stricto), and phototrophic euglenids are capable of rhythmic cellular deformations called "euglenoid movement" (syn. "metaboly") that is facilitated by adjacent strips sliding past one another at their zones of articulation [8-13]. Most phagotrophic euglenids have a robust feeding apparatus comprised of two main bundles of microtubules called "rods" and four interior "vanes" that are organized like a pinwheel in transverse section [9,14,15]. The feeding apparatus is highly developed in phagotrophic euglenids and highly reduced in osmotrophic and phototrophic euglenids [16,17].

Studies that have placed the morphological diversity of euglenids into a molecular phylogenetic context demonstrate that the mode of nutrition corresponds with general ultrastructural and behavioral traits [8,9]. Although it is expected that euglenid cells are capable of absorbing nutrients from the environment regardless of other abilities to acquire nutrients (e.g., phagotrophy or phototrophy), euglenids can be separated into four functional groups based on distinct nutritional modes: bacterivores (cells that usually feed on bacteria-relatively small particles), eukaryovores (cells that usually feed on microbial eukaryotes-relatively large particles), primary osmotrophs (cells that have lost a feeding apparatus and are limited to the absorption of nutrients), and phototrophs (cells capable of acquiring nutrients through photosynthesis). Bacterivores and eukaryovores are each paraphyletic, and the latter evolved from the former; primary osmotrophs and phototrophs are monophyletic, and each group evolved from eukaryovorous ancestors independently [8,9,16,18]. There are a couple of caveats to these designations: (1) the bacterivorous and eukaryovorous designations are not immune from exceptions (e.g., some *Petalomonas *species are relatively large and can consume yeast cells); (2) the loss of photosynthesis within phototrophic euglenids has led to several different lineages of "secondary" osmotrophs (cells that retain a non-photosynthetic plastid plus other ultrastructural features of phototrophic species; e.g., *Euglena longa*); and (3) the term "osmotrophy" refers to euglenid species that are limited to the absorption of nutrients and is not intended to imply that photosynthetic and phagotrophic euglenids are incapable of the absorbing nutrients from the environment.

Nonetheless, bacterivorous euglenids (e.g., *Petalomonas, Ploeotia *and *Entosiphon*) glide along substrates using one or two flagella, have a simple to complex feeding apparatus, and tend to be smaller with a rigid pellicle consisting of relatively few longitudinal strips (i.e., 4-12). Eukaryovorous euglenids (*Peranema, Heteronema *and *Urceolus*) also glide along substrates using one or two flagella, have a complex feeding apparatus, and tend to be larger with a flexible pellicle consisting of a relatively large number of helical strips (i.e., 16-60). The two main groups of euglenids that have lost phagotrophy, namely phototrophs and primary osmotrophs, contain lineages capable of euglenoid movement and several derived lineages that have lost euglenoid movement. The presence of euglenoid movement in some phototrophs and primary osmotrophs is inferred to reflect morphostasis from their eukaryovorous ancestors [8,9,13,18]. Primary osmotrophic euglenids swim in the water column using one or two flagella, lack a feeding apparatus, and have either a flexible or a fused pellicle consisting of a relatively large number of helical strips (i.e., usually more than 16-30, *Rhabdomonas costata *has 6-8). Phototrophic euglenids are separated into two main groups: the Eutreptiales and the Euglenales. Members of the Eutreptiales are largely marine, swim in the water column using two or more flagella, contain chloroplasts, lack a conspicuous feeding apparatus, possess a pronounced photoreception apparatus, and have a flexible pellicle consisting of a relative large number of helical strips (i.e.,16-50). Members of the Euglenales are largely freshwater, normally swim in the water column using one emergent flagellum, contain chloroplasts, lack a conspicuous feeding apparatus, possess a pronounced photoreception apparatus, and have a flexible or rigid pellicle consisting of a relatively large number of (usually) helical strips (i.e., 16-120). The pellicle of phototrophic euglenids can be distinguished from all other euglenids by the presence of "posterior whorls of strip reduction" (i.e., patterns of strips that terminate before reaching the posterior end of the cell). The actual number of pellicle strips and posterior whorls of strip reduction present on any particular cell reflects phylogenetic relationships and fundamental developmental mechanisms in euglenids [8-10,13,19].

The chloroplasts of phototrophic euglenids originated through a secondary endosymbiotic relationship between eukaryovorous euglenids and green algae. The pellicle and feeding apparatus in species like *Heteronema *and *Urceolus *best approximate the inferred features present in the most recent non-photosynthetic ancestor of phototrophic euglenids [8,13,16]; the chloroplasts of prasinophyceans like *Pyramimonas *best approximate the inferred features present in the most recent ancestor of all euglenid chloroplasts [20-22]. Nonetheless, there are still substantial differences between the morphological and behavioral features of eukaryovorous euglenids and phototrophic euglenids that limit our ability to fully portray the evolutionary transition between these two very different lifestyles. An improved understanding of euglenid diversity is expected to help bridge remaining gaps in our knowledge of this key event. In this vein, we have discovered and comprehensively characterized a novel marine euglenid with a uniquely "mixotrophic" lifestyle; this species contains a functional chloroplast and habitually consumes a specific strain of green algae. In this context, the term "mixotrophy" refers to a euglenid that is capable of both phagotrophy and phototrophy. Behavioral and ultrastructural data derived from high resolution light microscopy, scanning and transmission electron microscopy (SEM and TEM), and molecular phylogenetic analysis of small subunit (SSU) rDNA sequences demonstrate that this novel species conforms to the expected transitional stage between phototrophic euglenids and eukaryovorous ancestors.

## Methods

### Collection of organism and maintenance of cultures

Seawater samples were collected from a tide pool at Pachena Beach, Bamfield, British Columbia, Canada (48° 47.551' N, 125° 06.974' W) on June 18, 2010. The samples were inoculated in Provasoli's Enriched Seawater (PES) medium [23] and maintained at room temperature for one week. *Tetraselmis *sp. was isolated from the enrichment culture by micropipetting into sterile PES medium. *Rapaza viridis *n. gen. et sp. was also isolated from the enrichment culture into PES medium containing *Tetralselmis *sp. as a food source. Both cultures were incubated at 20°C under an illumination of 55-59 μmol photons/m^2^/s with 12:12 light:dark (L:D) light regime. The cultures were transferred every 10 to 14 days by bringing 1 ml of the culture into 25 ml of PES medium and prey cells. The cultures of *R. viridis *n. gen. et sp. and *Tetraselmis *sp. have been deposited into the American Type Culture Collection (ATCC), Manasas, VA, USA as PRA-360 and PRA-361, respectively.

### Light microscopy

Differential interference contrast (DIC) light micrographs were generated using a Zeiss Axioplan 2 imaging microscope equipped with Leica DC500 digital camera. Digital videos of live cells were generated using a Zeiss Axioplan 2 equipped with a Q imaging Microimager II digital camera and Q Capture v 2.8.1 software.

### Growth experiments

Cells of *R. viridis *were exposed to several different food sources in addition to *Tetraselmis *sp. ATCC PRA-361. Cultures of *Tetraselmis *sp. NEPCC365, *Tetraselmis *sp. NEPCC498, *T. striata *NEPCC487 and *T. tetrathelle *NEPCC 483 were obtained from the Canadian Center for the Culture of Microorganisms (CCCM) at the University of British Columbia, Vancouver, BC, Canada. A culture of *Navicula *sp. ATCC PRA-314 was obtained from the American Type Culture Collection (Manasas VA, USA). Cultures of the prasinophyte *Pycnococcus *sp., the chlorophyte *Dunaliella *sp., and the dinoflagellate *Scrippsiella trochoidea *were established in the lab from different marine environments independently.

Cultures of *R. viridis *were starved of prey cells and observed every 2-3 days until the death of all *R. viridis *cells was confirmed. We confirmed the existence of at least one chloroplast in the starved *R. viridis *cells using a Zeiss Axioplan 2 imaging microscope after one week, three weeks and five weeks from the start of starvation.

Cultures of *R. viridis *were also grown in the dark at 20°C. Prey cells (*Tetraselmis *sp., ATCC PRA-361) were added every 1-2 days until the death of all *R. viridis *cells was confirmed. As a control experiment, cultures grown under normal light conditions were also treated exactly like the cultures grown in the dark (e.g., the same volume of prey cells was added at the same intervals of time).

### Scanning electron microscopy

A culture of *R. viridis *was mixed in 1% (v/v) OsO_4 _in seawater at room temperature. The fixed cells were mounted on polycarbonate Millipore filters (13 mm diam., 5 μm pore size) or glass plates coated with poly-L-lysine at room temperature for 15 min. The cells were rinsed with distilled water and dehydrated with a graded ethanol series from 30% to absolute ethanol before being critical point dried with CO_2 _using a Tousimis Critical Point Dryer. The dried cells were then coated with gold using a Cressington 208HR High Resolution Sputter Coater and observed with a Hitachi S-4700 field emission SEM.

### Transmission electron microscopy

Two different cultures were examined with transmission electron microscopy (TEM): (1) a culture starved of *Tetraselmis *sp. cells for three weeks and (2) a culture that was fed cells of *Tetraselmis *sp. one hour prior to fixation. The cultures were pre-fixed in 2.5% (v/v) glutaraldehyde with 0.2 M sucrose in 0.1 M sodium cacodylate buffer (SCB) (pH 7.2) at room temperature for 2 hours. The pre-fixed cells were washed in 0.2 M SCB (pH 7.2) twice and post-fixed in 1% (w/v) osmium tetroxide in 0.2 M SCB (pH 7.2) at room temperature for 1 hour. The fixed cells were dehydrated through a graded series of ethanol and 100% acetone. The dehydrated cells were then infiltrated with acetone-Epon 812 resin mixtures and 100% Epon 812 resin. Ultra-thin serial sections were collected on copper Formvar-coated slot grids, stained with 2% (w/v) uranyl acetate and lead citrate, and observed using a Hitachi H7600 TEM.

### DNA extraction, PCR amplification, alignment and phylogenetic analysis

Genomic DNA was extracted using the MasterPure Complete DNA and RNA purification Kit (Epicentre, WI, USA) from a culture of *R. viridis *that was starved of prey cells for three weeks. Polymerase chain reactions (PCR) were performed using PuRe Taq Ready-To-Go PCR beads kit (GE Healthcare, Buckinghamshire, UK). The nearly complete eukaryotic SSU rDNA gene was amplified using the eukaryotic universal primers 5'-TGATCCTTCTGCAGGTTCACCTAC-3' and 5'-GCGCTACCTGGTTGATCCTGCCAGT-3' with the same PCR protocol described by Breglia et al. (2010) [24]. The amplified DNA fragments were purified from agarose gels using UltraClean 15 DNA Purification Kit (MO Bio, CA, USA), and subsequently cloned into the TOPO TA Cloning Kit (Invitrogen, CA, USA). One clone was sequenced using ABI Big-Dye reaction mix (BigDye 3.1) using the vector forward and reverse primers and also internal primers (nomet1134R: 5'-TTTAAGTTTCAGCCTTGCG-3' and SR4Eug: 5'-ACTGGAGGGCAAGYCTGGT-3') oriented in both directions. The new sequence was initially identified by BLAST analysis, confirmed with molecular phylogenetic analysis, and deposited into GenBank: AB679269.

The SSU rRNA sequence from *R. viridis *was added to a 39-taxon alignment focused on the diversity of euglenids using representative kinetoplastids and diplonemids as an outgroup. Ambiguously aligned positions and gaps were excluded from alignment, leaving 805 unambiguously aligned positions; the alignment is available from the authors upon request.

Maximum likelihood (ML) analysis was performed on the 39-taxon alignment using PAUP* version 4.0b10 [25]. Prior to starting the ML analysis, we used Akaike information criterion (AIC) test as implemented in the software jModelTest 0.1.1. [26] to find the model of evolution that best fits the data set for the Maximum likelihood (ML) analysis. The result indicated that the TIM1ef, which allows for equal base frequencies and four substitution rates (AC = GT; AT = CG; AG, CT) [27,28], plus gamma model should be used for this data set. The parameters were as follows: assumed nucleotide frequencies are equal; substitution rate matrix with A-C substitutions = 1.0000, A-G = 2.7002, A-T = 0.7141, C-G = 0.7141, C-T = 3.8573, G-T = 1.0000; proportion of sites assumed to be invariable = 0 and rates for variable sites assumed to follow a gamma distribution with shape parameter = 0.3930. The ML tree was implemented using the heuristic search option with TBR branch swapping. Bootstrap analyses [29] were carried out for ML with 500 replicates to evaluate statistical reliability.

The alignment was also analyzed with Bayesian methods using the MrBayes 3.1.2 [30]. The program was set to operate the GTR model with a gamma distribution and four Monte-Carlo-Markov chains (MCMC) starting from a random tree. A total of 500,000 generations were calculated with trees sampled every 100 generations. The first 1,250 trees in each run were discarded as burn-in using the sumt command. Posterior probabilities correspond to the frequency at which a given node was found in the post burn-in trees.

### Sequence availability

The SSU rDNA nucleotide sequences included in 39-taxon analyses for this paper are available from the GenBank database under the following accession numbers: *Anisonema acinus *[GenBank:AF403160], *Bihospites bacati *[GenBank:HM004354], *Bodo saltans *[GenBank:AY998648], *Calkinsia aureus *[GenBank:EU753419], *Colacium *sp. [GenBank:DQ140154], *Dimastigella mimosa *[GenBank:DQ207576], *Dinema sulcatum *[GenBank:AY061998], *Diplonema ambulator *[GenBank:AF380996], *Diplonema papillatum *[GenBank:AF119811], *Discoplastis spathirhyncha *[GenBank:AJ532454], *Distigma proteus *[GenBank:AF106036], *Entosiphon *sp. [GenBank:AY425008], *Entosiphon sulcatum *[GenBank:AF220826], *Euglena gracilis *[GenBank:AF283308], *Euglena longa *(as *Astasia longa*) [GenBank:AF112871], *Euglena quartana *(as *Khawkinea quartana*) [GenBank:U84732], *Euglena stellata *[GenBank:AF081590], *Euglena viridis *[GenBank:AF445460], *Eutreptia viridis *[GenBank:AF157312], *Eutreptiella gymnastica *[GenBank:AF081590], *Eutreptiella pomquetensis *[GenBank:AJ532398], *Ichthyobodo necator *[GenBank:AY224691], *Lepocinclis buetschlii *[GenBank:AF096993], *Menoidium cultellus *[GenBank:AF295019], *Monomorphina *sp. [GenBank:DQ140130], *Neobodo designis *[GenBank:AF209856], *Notosoleus ostium *[GenBank:AF403159], *Peranema *sp. [GenBank:AY048919], *Peranema trichophorum *[GenBank:AF386636], *Petalomonas cantuscygni *[GenBank:AF386635], *Phacus aenigmaticus *[GenBank:AF283313], *Ploteotia costata *[GenBank:AF525486], *Rapaza viridis *[GenBank: AB679269], *Rhabdomonas costata *[GenBank:AF295021], *Rhynchomonas nasuta *[GenBank:AY998642], *Rhynchopus *sp. [GenBank:AF380997], *Strombomonas triquetra *[GenBank:DQ140153], *Trypanosoma evansi *[GenBank:AY904050], *Trypanosoma *sp. [GenBank:EF375883].

### Archiving

A digital archive of this paper is available from PubMed Central and print copies are available from libraries in the following five museums: Natural History Museum Library (Cromwell Road, London, SW7 5BD, UK), American Museum of Natural History (Department of Library Services, Central Park West at 79th St., New York, NY, 10024, USA), Muséum national d'Histoire naturelle (Direction des bibliothèques et de la documentation, 38 rue Geoffroy Saint-Hilaire, 75005 Paris, France), Russian Academy of Sciences (Library for Natural Sciences of the RAS Znamenka str., 11, Moscow, Russia) and Academia Sinica (Life Science Library, 128 Sec. 2 Academia Rd, Nankang Taipei 115, Taiwan R.O.C.).

## Results

### General morphology

**Figure 1:**
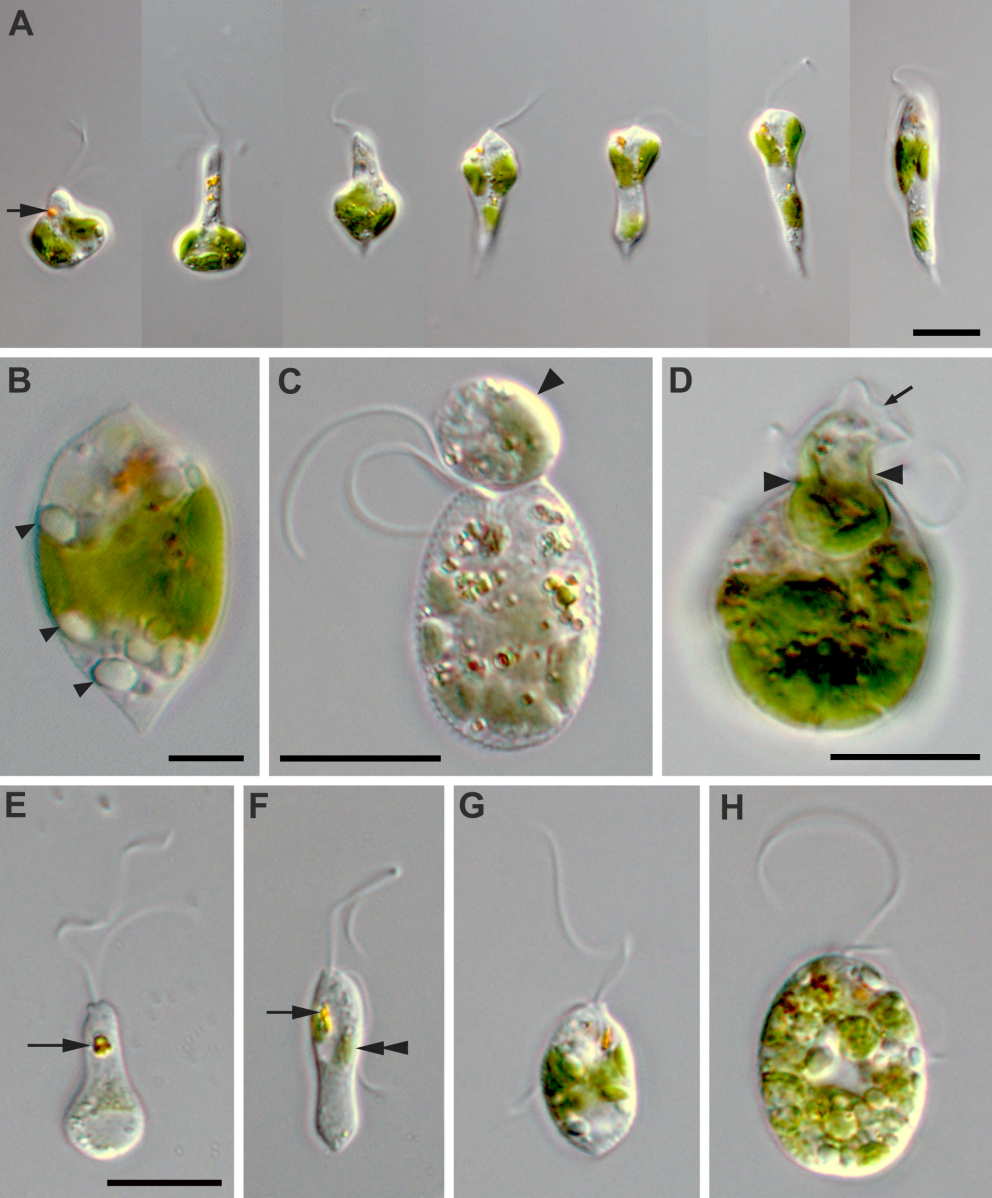
**Differential interference contrast (DIC) light micrographs (LM) of *Rapaza viridis *n. gen. et sp. showing general morphological features of the species**. **A**. Time series of a live cell showing euglenoid movement (arrow, stigma). **B**. LM showing paramylon grains (arrowheads) free in the cytoplasm. **C**. LM showing *R. viridis *capturing a *Tetraselmis *cell (arrowhead) with the anterior part of the cell. **D**. LM showing *R. viridis *engulfing a *Tetraselmis *cell. The arrow points to the theca of *Tetraselmis*, and the arrowheads denote the expanded cytostome of *R. viridis*. **E-F**. LMs of *R. viridis *cells that have been starved for one week (arrow, stigma; double arrowhead, euglenid chloroplast). **G-H**. LMs of *R. viridis *cells that were fed 24 hours earlier. **E-H**. same magnification. Scale bars 10 μm in **A**, **C**-**H**; 5 μm in **B**.

**Figure 2:**
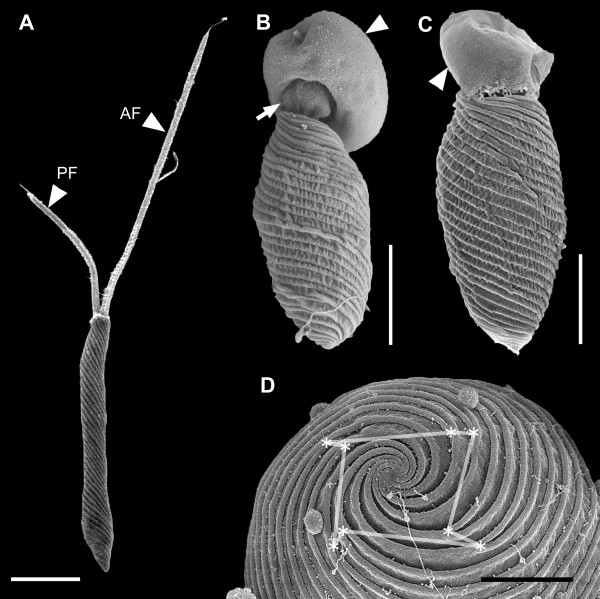
**Scanning electron micrographs (SEM) of *Rapaza viridis *n. gen. et sp**. **A**. SEM showing a relaxed elongated cell with helical pellicle strips and two flagella. The anterior flagellum (AF) and the posterior flagellum (PF) insert into the anterior part of the cell. **B**. SEM showing an *R. viridis *cell that has bored a hole into the cell surface of a captured *Tetraselmis *cell (arrowhead). The internal contents of the captured *Tetraselmis *cell (arrow) are being withdrawn by myzocytosis. **C**. SEM showing *R. viridis *engulfing a *Tetraselmis *cell (arrowhead). **D**. SEM of *R. viridis *showing posterior strip reduction (*), which connected by lines, forms a square-shaped whorl. Scale bars 5 μm in **A**-**C**; 2 μm in **D**.

**Figure 3:**
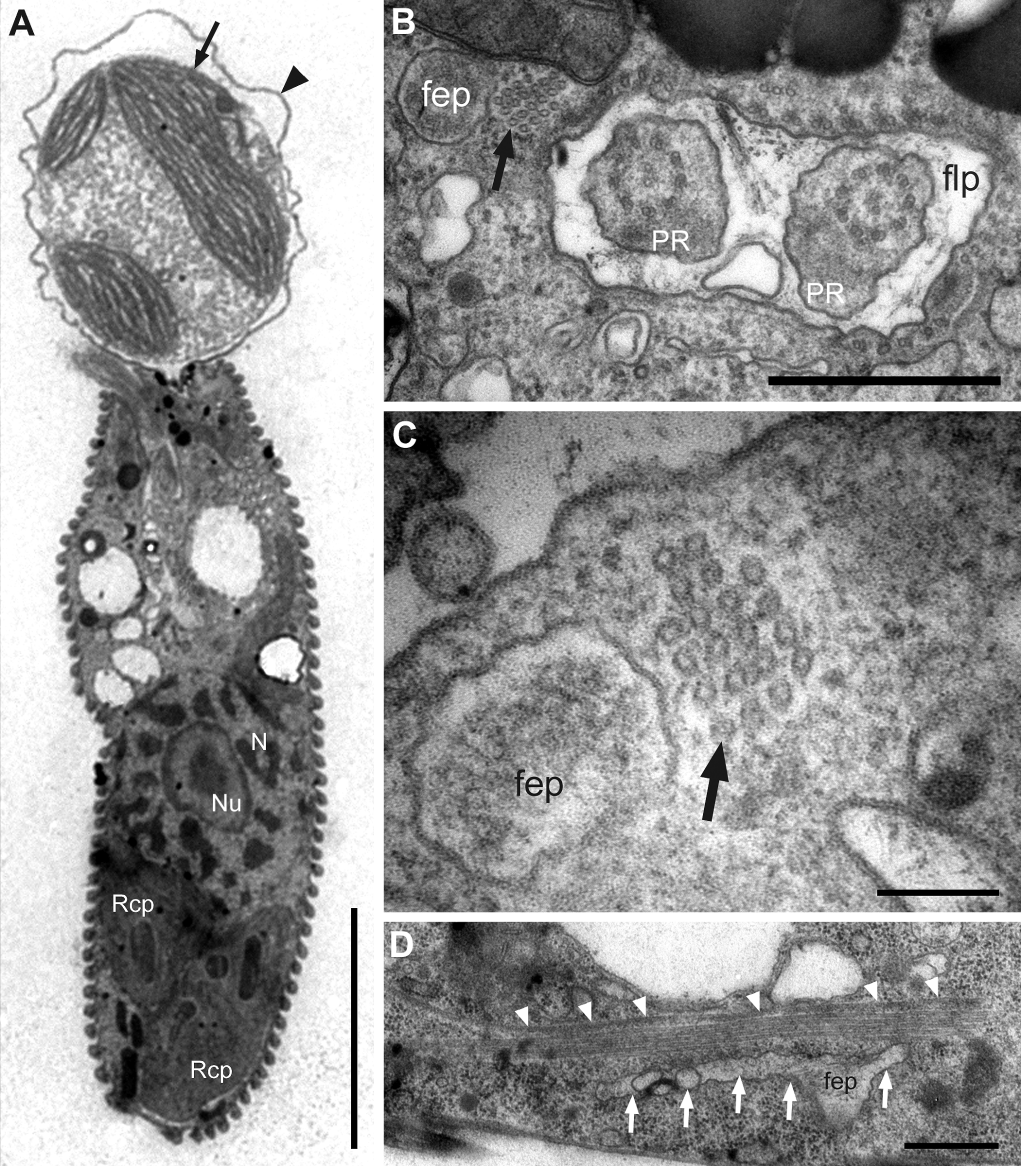
**Transmission electron micrographs (TEM) of *Rapaza viridis *n. gen. et sp. showing details of the feeding apparatus**. **A**. Longitudinal TEM showing *R. viridis *capturing a *Tetraselmis *cell (arrow). The arrowhead indicates the theca (cell wall) of *Tetraselmis*, which is beginning to unravel. The chloroplast of *R. viridis *(Rcp) and the nucleus (N) containing permanently condensed chromosomes and a large nucleolus (Nu) is also visible. **B**. Transverse TEM through the flagellar pocket (flp) showing paraxial rods (PR), the pocket (fep) and the microtubules of the feeding rod (arrow). **C**. Transverse TEM through the feeding rod showing 20 microtubules organized in four rows (4-6-6-4). **D**. Longitudinal TEM through the feeding apparatus showing the feeding pocket ('fep' and arrows) and microtubules of the rod (arrowheads). Scale bars 5 μm in **A**; 1 μm in **B **and **D**; 200 nm in **C**.

**Figure 4:**
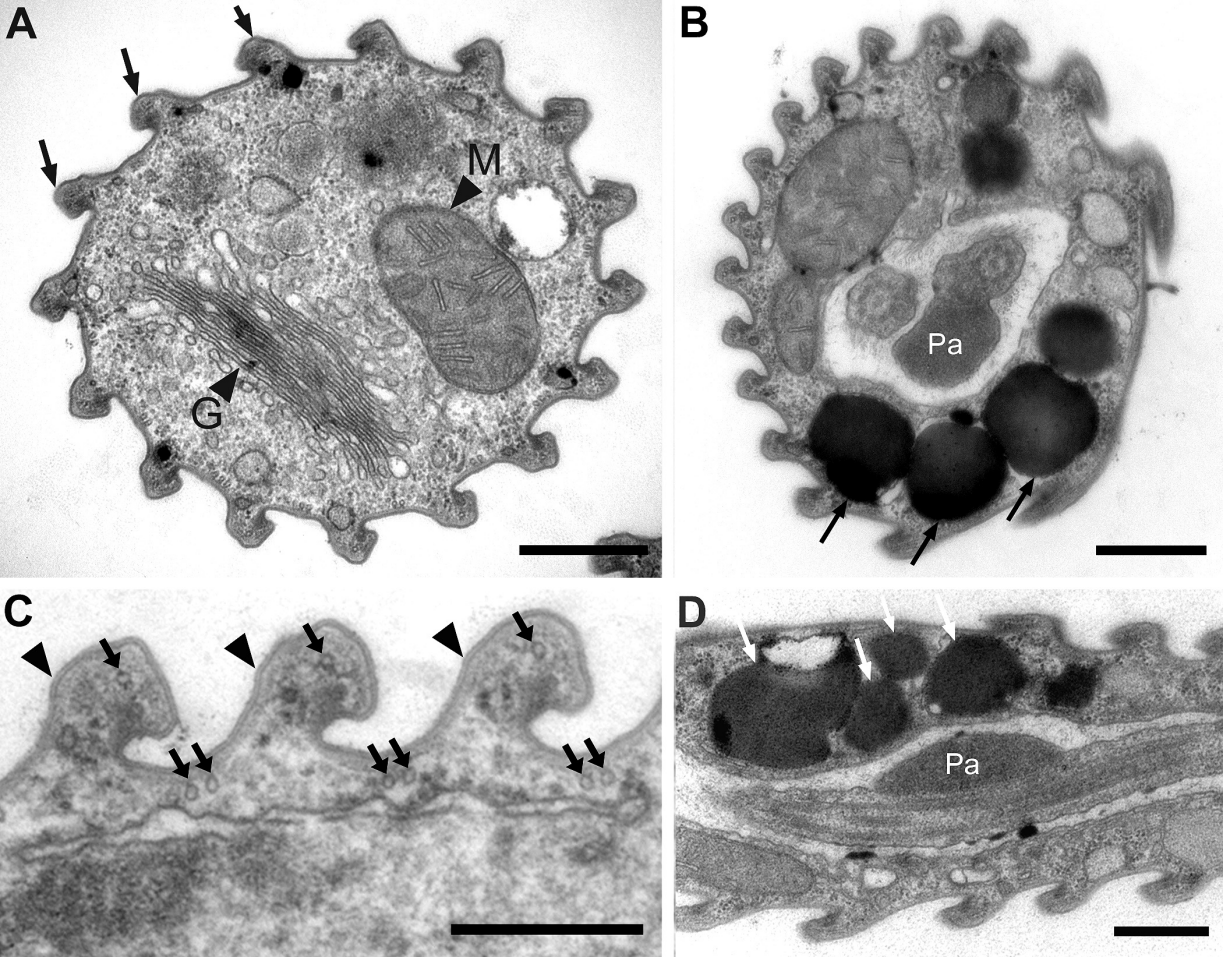
**Transmission electron micrographs (TEM) of *Rapaza viridis *n. gen. et sp. showing details of the cytoplasm, pellicle and photoreception apparatus**. **A**. Transverse TEM showing the mitochondrion (M-arrowhead), the Golgi body (G-arrowhead) and 16 pellicle strips (arrows). **B**. Transverse TEM showing the stigma (arrows) and the paraflagellar swelling (Pa). **C**. Transverse TEM showing the S-shaped frames of the pellicle strips (arrowheads) and underlying microtubules (arrows). **D**. Sagittal TEM showing the stigma (arrows) and the paraflagellar swelling (Pa). Scale bars 1 μm in **A**, **B **and **D**; 500 nm in **C**.

**Figure 5:**
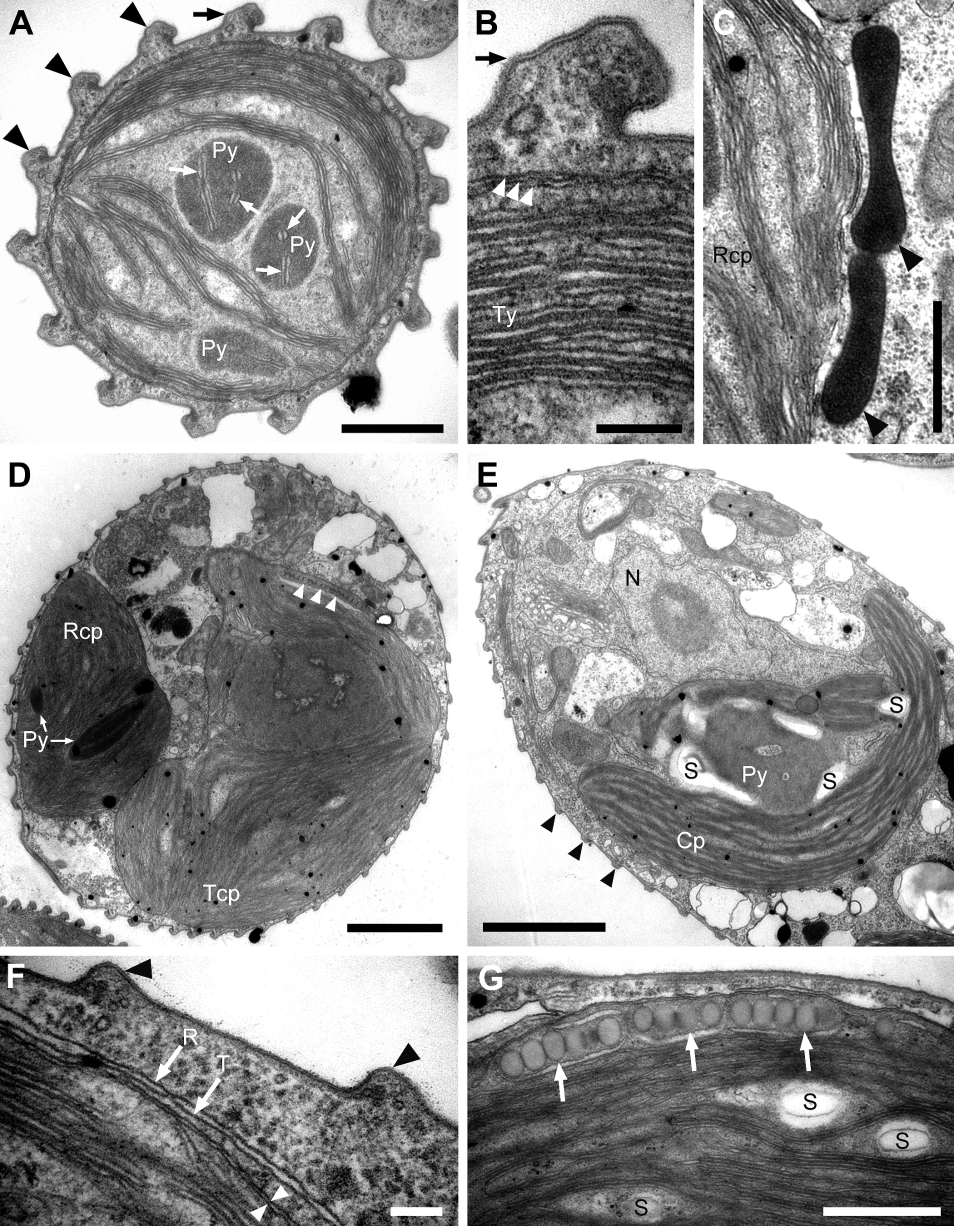
**Transmission electron micrographs (TEM) of *Rapaza viridis *n. gen. et sp. showing details of two types of chloroplasts**. **A**. Transverse TEM showing the chloroplast of *R. viridis *and 16 pellicle strips (arrowheads). Pairs of thylakoid membranes (arrows) penetrate the pyrenoids (Py). The black arrow indicates the the pellicle strip that is shown in Figure 5B. **B**. Higher magnification TEM of Figure 5A focusing on the three membranes that surround the chloroplast of *R. viridis *(arrowheads) and the associated stacks of thylakoids (Ty). The black arrow indicates the the pellicle strip that is shown in Figure 5A. **C**. High magnification TEM showing electron-dense structures surrounded by a single membrane (arrowheads) that are situated near a chloroplast. **D**. TEM showing two different types of chloroplasts: The chloroplast of *Tetraselmis *sp. (Tcp) contains an eyespot (arrowheads); the chloroplast of *R. viridis *(Rcp) does not. **E**. Longitudinal TEM showing an engulfed *Tetraselmis *cell, including the nucleus (N), pyrenoid (Py) and associated starch (S) and chloroplast (Cp). Arrowheads indicate the pellicle strips of *R. viridis*. **F**. High magnification TEM showing the food vacuole of *R. viridis *(R-arrow) and the cell membrane of the engulfed *Tetraselmis *cell (T-arrow). White arrowheads mark the two membranes that surround the chloroplast of *Tetraselmis *sp. Black arrowheads mark the pellicle strips of *R. viridis*. **G**. High magnification TEM showing the eyespot (arrows) within the chlorplasts of *Tetraselmis *sp. Scale bars 1 μm in **A**, **C **and **G**; 200 nm in **B **and **F**; 4 μm in **D **and **E**.

The relaxed, (well-fed) swimming cells of *Rapaza viridis *n. gen. et sp. were oval, 10.5-38.2 μm long (mean ± SD = 19.5 ± 7.3 μm, n = 50), and 2.9-15.1 μm wide (mean ± SD = 8.4 ± 3.3 μm, n = 50). The cells of *R. viridis *usually swam in a spiral pattern with the flagella moving rapidly and chaotically. Euglenoid movement was also observed when the cells were feeding on *Tetraselmis *or when the cells were pressured by a cover glass (Figure 1A, Additional file 1). Two flagella emerged from the flagellar pocket, were unequal in length, were adorned with hairs, and contained heteromorphic paraxial rods (Figures 1E, 2A and 3B). The longer anterior flagellum was about 1.25 times the length of the cell in the relaxed state and always directed forward. The shorter posterior flagellum was about 0.65 times the length of the relaxed cell and directed backward; this flagellum was sometimes directed forward and moved oar-like (Additional file 1). A robust anterior stigma that was independent of the chloroplasts was comprised of one to more than ten particles ranging from 0.8-1.3 μm in diam. (Figures 4B and 4D). A paraflagellar swelling formed a dense, lens-like body that was positioned within the flagellar pocket and against the stigma (Figure 4D). The cytoplasm contained several ellipsoid paramylon grains (Figure 1B), large Golgi bodies, and mitochondria with discoidal (paddle-shaped) cristae (Figure 4A). The typical euglenid nucleus contained permanently condensed chromosomes and a large central nucleolus (Figure 3A). The pellicle consisted of 16 helically arranged strips (Figures 4A and 5A) with delicate S-shaped frames that were supported by underlying microtubules (Figure 4C). Eight pellicle strips terminated before reaching the posterior end of the cell; these strips were organized in pairs that formed the four corners of a square-like pattern when the terminating strips were traced with lines (Figure 2D). The feeding apparatus consisted of a feeding pocket and one adjacent feeding rod comprised of 20 microtubules organized in four rows (4-6-6-4) (Figures 3B, C and 3D). We did not observe any vanes or amorphous material associated with these microtubules (Figure 3).

### Feeding behavior

*R. viridis *ingested *Tetraselmis *cells when plentiful in the culture and were distinctly larger and bright green in the presence of food (Figures 1G and 1H). The *Tetraselmis *cells, including their chloroplasts, were completely digested over the course of about 12 hours. When starved of *Tetraselmis *prey, the cells of *R. viridis *became smaller and colorless, except for the retention of at least one intact chloroplast within the cytoplasm (Figures 1E and 1F). *R. viridis *could not survive for more than 35 days without exposure to a specific strain of *Tetraselmis *sp. (PRA-361). Different strains of *Tetraselmis *spp. and several other species of microalgae were added to the cultures in an attempt to grow *R. viridis *on an alternate food source: the prasinophytes *Tetraselmis *sp. NEPCC365, *Tetraselmis *sp. NEPCC498, *T. striata *NEPCC487, *T. tetrathelle *NEPCC 483, *Pycnococcus *sp., the diatom *Navicula *sp. ATCC PRA-314, the chlorophyte *Dunaliella *sp., and the dinoflagellate *Scrippsiella trochoidea. R. viridis *rejected all of these prey choices.

Adding *Tetraselmis *sp. (PRA-361) into a culture of starved *R. viridis *cells triggered a feeding frenzy. *R. viridis *captured *Tetraselmis *sp. (PRA-361) with the anterior part of the cell and either rotated rapidly or swam backward in a spiral pattern while dragging the prey cell (Figures 1C and 3A). Euglenoid movement was prominent during the processes of ingestion (Figures 1D, 2C and Additional file 2). *R. viridis *was capable of completely engulfing prey cells. In the process of engulfment, the theca (cell wall) of *Tetraselmis *was gradually loosened and peeled away by the repetitive peristaltic actions of euglenoid movement (Figures 1D and 2B-C). However, *Tetraselmis *cells with an intact theca could also be engulfed, but the theca was discharged from the anterior end of *R. viridis *cells soon afterwards. The continuous actions of euglenoid movement facilitated the complete ingestion and posterior transport of an engulfed *Tetraselmis *cell (Additional file 2). The entire process of engulfment took 5-40 minutes; at this point, the *R. viridis *cell would slow down euglenoid movement, elongate, and begin to swim again. Cells of *R. viridis *exposed to an ample food supply contained several ingested cells of *Tetraselmis *at one time.

### The chloroplasts of *Rapaza viridis *and *Tetraselmis *sp

There were two ultrastructurally distinct types of chloroplasts present in well-fed cells of *R. viridis*: (1) chloroplasts corresponding to the ultrastructure of green algae (i.e., *Tetraselmis*) and (2) chloroplasts corresponding to the ultrastructure of phototrophic euglenids. The euglenid-type chloroplasts were surrounded by three membranes, contained thylakoids in stacks of three, and contained one to three darkly stained pyrenoids without associated paramylon granules (Figures 5A, B and 5D). Double stacks of thylakoids also penetrated the pyrenoids in the euglenid-type chloroplasts (Figures 5A and 5D). Electron-dense structures surrounded by a single membrane were often observed just outside the euglenid chloroplast (Figure 5C). The *Tetraselmis*-type chloroplasts were often discernible within the cytoplasm of *R. viridis *by light microscopy because of their intraplastidial eyespots. The *Tetraselmis*-type chloroplasts were also distinguishable from the euglenid-type chloroplasts with TEM. A complete membrane surrounded engulfed *Tetraselmis *cells (Figure 5F). The pyrenoids in the *Tetraselmis*-type chloroplasts were surrounded by starch grains and did not contain penetrating thylakoids (Figure 5E). Two membranes, rather than three, surrounded the *Tetraselmis*-type chloroplasts (Figure 5F). Moreover, unlike the euglenid-type chloroplasts, intraplastidial eyespot globules were easily seen at the periphery of *Tetraselmis*-type chloroplasts (Figures 5D and 5G).

Starved cells of *R. viridis *almost always contained at least one intact chloroplast (Figures 1E and 1F). TEM demonstrated that this enduring chloroplast had the euglenid-type ultrastructure (Figure 5A). When cells of *R. viridis *were grown in the absence of light, they could not survive for more than one week. The result of this dark growth experiment was consistent even in the presence of an ample and continuous supply of *Tetraselmis *prey cells. Cells of *R. viridis *required both photosynthesis and (*Tetraselmis*) prey cells in order to survive.

### Molecular phylogenetic position

**Figure 6:**
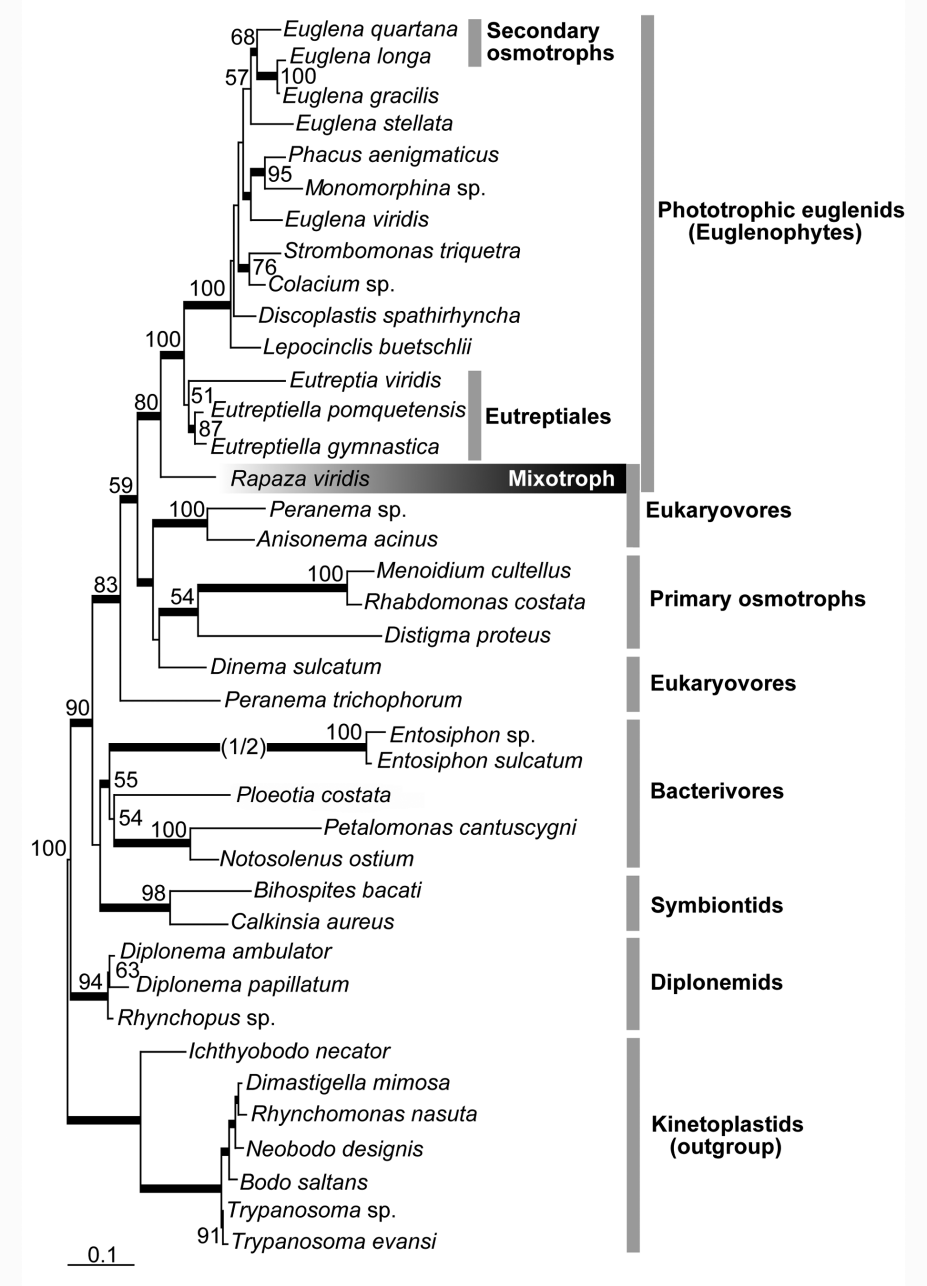
**Phylogenetic position of *Rapaza viridis *n. gen. et sp. within the Euglenozoa as inferred from SSU rRNA gene sequences**. Maximum-likelihood (ML) tree constructed using TIM1ef + G model of evolution on an alignment of 39 taxa and 805 unambiguously aligned sites, including 298 constant positions and 432 informative positions, using seven kinetoplastid and three diplonemid sequences as the outgroup. ML bootstrap values greater than 50% are shown. Thick branches indicate Bayesian posterior probabilities (PP) over 0.95. The branch leading to the fast-evolving *Entosiphon *clade has been shortened by one half (indicated by '1/2').

We determined the nearly complete sequence of the SSU rRNA gene of *R. viridis *(2,669 bp). Molecular phylogenetic analysis of the 39-taxon alignment demonstrated that phototrophic euglenids (syn. euglenophytes), including secondary osmotrophic species (*Euglena longa *and *E. quartana*), formed a robust monophyletic group (100% bootstrap value and 1.00 Bayesian posterior probability) (Figure 6). *R. virdis *formed the nearest sister lineage to clade of phototrophic euglenids with relatively high statistical support (80% bootstrap value and 1.00 Bayesian posterior probability).

## Discussion

Along with all of the expected euglenid features (e.g., pellicle strips, mitochondria with discoidal cristae, a nucleus with permanently condensed chromosomes, and paraxial rods), *R. viridis *possessed two heterodynamic flagella (rather than one emergent flagellum), swimming behavior, and euglenoid movement that was most reminiscent of members within the Eutreptiales. Like most species of *Eutreptia *and *Eutreptiella, R. viridis *lives in marine environments, which stands in contrast to the freshwater lifestyles of the vast majority of species within the Euglenales (e.g., *Euglena, Phacus, Lepocinclis *and *Trachelomonas*). However, *R. viridis *is clearly distinct from members of the Eutreptiales because of its eukaryovorous behavior and associated feeding apparatus. Moreover, the molecular phylogenetic analyses of SSU rDNA sequences supports the placement of *R. viridis *as the nearest sister lineage to all photosynthetic euglenids (i.e., the clade consisting of the Eutreptiales and the Euglenales) rather than sister to or within the Eutreptiales. *R. viridis *is also clearly distinct from other eukaryovorous euglenids in having features present in all other phototrophic euglenids, such as functional chloroplasts and a robust plastid-independent photoreception apparatus (i.e., a stigma plus a photosensory swelling at the base of the dorsal flagellum). Although there is inconclusive evidence that the eukaryovore *Urceolus cyclostomus *might also possess a weakly developed photoreception apparatus [16], *R. viridis *represents the first indisputable example of a eukaryovorous euglenid with photosensory ability. This would enable *R. viridis *to maintain its position in the water column in order to increase the likelihood of encountering its preferred photosynthetic prey cells (*Tetraselmis *sp.). However, because *R. viridis *is also photosynthetic, the photosensory ability could have more to do with maintaining an optimal position in the light for its own photosynthesis. Overall, the molecular phylogenetic position and combination of morphological and behavioral traits in *R. viridis *is more than just novel; these data completely conform to the inferred ancestral traits that existed during the evolutionary transition from eukaryovorous lifestyles to phototrophic lifestyles.

### Transitional character states

**Figure 7:**
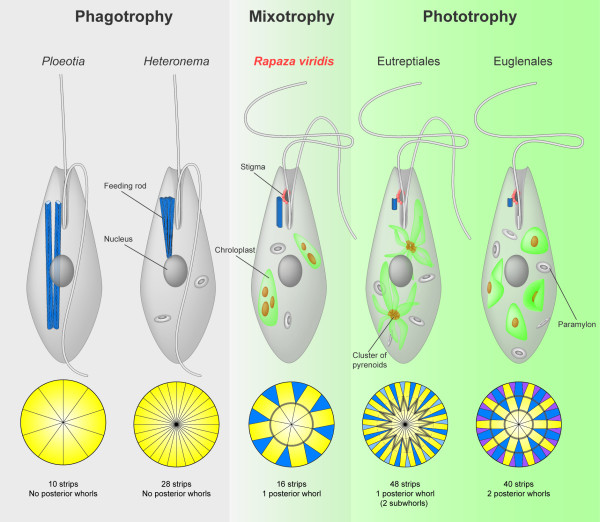
**Illustrations showing the transitional character states present in *Rapaza viridis *n. gen. et sp. that link phagotrophic euglenids with phototrophic euglenids**. Illustrations of five different euglend cells emphasizing the size of the feeding apparatus, the number of flagella, and the presence or absence of chloroplasts, pyrenoids, paramylon grains and a stigma. The illustrations below each representative cell show the number of pellicle strips and whorls of posterior strip reduction as indicated by different colors. Pellicle strips that reach the posterior end of the cell are shown in yellow; strips that terminate before reaching the posterior end are shown in blue and purple. The solid grey lines that connect the terminating strips represent each exponential whorl of reduction. Note that the cell representing the Eutreptiales has one zigzag (exponential) whorl associated with two staggered subwhorls. The illustrations are based on the ultrastructural studies of *Ploeotia *[45-47], *Heteronema scaphurum *[48], the Eutreptiales (e.g., *Eutreptia pertyi*) [14,19,36,49], and the Euglenales (e.g., *Euglena laciniata *for the pattern of pellicle strips and *Eugelna gracilis *for general shield-shaped plastids) [19,50,51].

The molecular phylogenetic analysis of SSU rDNA placed *R. viridis *squarely between phagotrophic and phototrophic euglenids as the nearest sister lineage to the entire euglenophyte clade (i.e., all phototrophic euglenids and their secondary osmotrophic descendants). This phylogenetic context alone makes *R. viridis *a particularly intriguing candidate for gaining new insights into euglenid character evolution, especially in regard to the endosymbiotic origin of chloroplasts. The ultrastructural and behavioral features in *R. viridis *were perhaps even more compelling because they were so intermediary between the features already described in phagotrophic and phototrophic euglenids (Figure 7); this is especially relevant for characters associated with modes of nutrition, the feeding apparatus, the flagellar apparatus and the pellicle.

#### Mixotrophy

*R. viridis *was unable to survive when placed in the dark or when deprived of *Tetraselmis *prey cells; therefore, *R. viridis *required both photosynthesis and phagotrophy to satisfy its overall nutritional requirements. This "mixotrophic" mode of nutrition is rare and has only been described in a few groups of eukaryotes, such as in some dinoflagellates, haptophytes and stramenopiles [31-33]. Until now, mixotrophy was completely unknown within the Euglenida. Nonetheless, a mixotrophic mode of nutrition is a logical transitional stage during the establishment of endosymbiotic partnerships between (ancestrally) phagotrophic host cells and phototrophic prey cells (Figure 7). Once the required horizontal gene transfer events and gene retargeting mechanisms established a permanent photosynthetic organelle within a new host cell, the ability to lose phagotrophy and to rely exclusively on phototrophy becomes possible [34,35]. This has clearly happened in several different lineages of eukaryotes independently, including in the most recent ancestor of all phototrophic euglenids [8]. Therefore, the mixotrophic mode of nutrition in *R. viridis *is entirely consistent with the molecular phylogenetic position of this species and clarifies an important transitional stage in the origin of phototrophy in euglenids (Figure 7).

#### Feeding apparatus

The main components of the feeding apparatus in most phagotrophic euglenids are two robust rods that can extend the entire length of the cell. Phototrophic euglenids have retained a highly reduced feeding apparatus without rods [9,17]. A few phototrophic euglenids within the Eutreptiales, however, have retained small clusters of microtubules that are inferred to be homologous to the rods of phagotrophic euglenids [36,37]. The feeding rod we characterized in *R. viridis *is intermediate in size and organization between the robust rods in phagotrophic species and the reduced rods in the Eutreptiales (e.g., *Eutreptiella eupharyngea*) (Figure 7). This result is entirely consistent with the molecular phylogenetic position of this species, substantiates the above homology statement, and clarifies an important transitional stage in the origin of phototrophy in euglenids (Figure 7).

#### Flagellar apparatus

Phagotrophic euglenids generally glide along surfaces with two relatively thick heterodynamic flagella: the anterior (dorsal) flagellum is directed forward and twitches at the tip; the posterior (ventral) flagellum is directed backwards and glides against the substrate [8]. Phototrophic (and osmotrophic) euglenids generally swim in the water column using one or two flagella that move somewhat erratically or move in a controlled figure-eight configuration that pulls the cell forward. These modes of swimming allow phototrophic euglenids to exploit the water column above the substrate [9]. The cells of *R. viridis *were capable of swimming in the water column in a pattern that was most reminiscent of species within the Eutreptiales. Therefore, *R. viridis *represents the first example of a eukaryovorous euglenid that is also capable of swimming in the water column; this is another feature that is consistent with the molecular phylogenetic position of this species and that clarifies an important transitional stage in the origin of phototrophy in euglenids (Figure 7).

#### Pellicle

The substructure, number, and organization of pellicle strips reflects phylogenetic relationships within euglenids [8-11,13,19]. For instance, members of the Euglenales tend to have strips with relatively thick proteinaceous frames and underlying projections that interconnect adjacent strips [9,11,13,38]. Phagotrophic species, (plastic) primary osmotrophic species, and members of the Eutreptiales have relatively thin proteinaceous frames without projections. The strip substructure of *R. viridis *was most similar to the strips in members of the Eutreptiales and eukaryovores like *Dinema *and *Peranema*, which is consistent with the transitional state expected between eukaryovorous and phototrophic euglenids [11,13]. The total number of strips in *R. viridis *(16) was relatively low in comparison to *Peranema *and *Eutreptia *and more consistent with the number of strips found in eukaryovores like *Dinema *and primary osmotrophs like *Distigma *(16-22) [10,16].

Posterior whorls of strip reduction have only been observed in phototrophic euglenids; however, some relatively obscure patterns of posterior strip reduction have also been observed in one eukaryovore (*Peranema*; [13]). The number and pattern of posterior whorls of strip reduction varies in different species and reflects phylogenetic relationships within phototrophic euglenids [19,39]. Posterior whorls of strip reduction are usually "exponential" (i.e., the number of strips that terminate on a whorl equals the number of strips that pass through the whorl, leading to a pattern whereby the number of strips is halved at each whorl), but in some species the whorls are separated into two or more "linear" subwhorls (i.e., the number of strips that terminate is the same for all subwhorls; Figure 7) [10,13,39]. The range for the number of exponential whorls of strip reduction known so far in the Euglenales is two to four [19]. One member of the Eutreptiales (*Eutreptia pertyi*) has been shown to possess two linear subwhorls of posterior strip reduction that corresponds to one exponential whorl of strip reduction [13] (Figure 7). Aside from a few derived species of *Phacus *[40], *R. viridis *is the first and only euglenid described so far with one clear exponential whorl of strip reduction; this precisely corresponds to the expected character state that was present in the most recent ancestor of all euglenids possessing posterior whorls of strip reduction (i.e., all phototrophic species) [9,13,19]. The eight strips that terminate on the whorl of strip reduction in *R. viridis *form four distinct pairs that, when connected by lines, define the equidistant corners of a square (Figure 2D). This pattern of posterior strip reduction is novel among all euglenids characterized so far.

### The chloroplasts of *Rapaza viridis*

Some dinoflagellates, animals, and foraminiferans possess "kleptochloroplasts", which are transiently functional chloroplasts derived from algal food. The host cells retains these chloroplasts for a short period of time in order to obtain products of photosynthesis as a source of nutrition; hosts are not capable of maintaining chloroplasts as permanent organelles that can be inherited from generation to generation. Kleptochloroplasts are ultimately digested, and the host organism needs to replenish them by regularly consuming algal food. Eukaryovorous euglenids (e.g., *Peranema *and *Urceolus*) do not have chloroplasts but they often engulf microalgae as their primary source of food, so well-fed cells can superficially appear full of chloroplasts; these chloroplasts disappear when the eukaryovorous cell is starved. By contrast, the cells of *R. viridis *always retained at least one chloroplast even when starved for over a month and cannot survive in the dark even when regularly exposed to an abundant food supply. Therefore, cells of *R. viridis *require both photosynthesis and (*Tetraselmis*) prey cells in order to survive.

Our ultrastructural observations of well-fed and starved cells of *R. viridis *demonstrated that this species usually contains both stable chloroplasts and transient *Tetraselmis *chloroplasts that can be readily distinguished from one another. The stable chloroplasts in *R. viridis *were surrounded by three membranes, lacked an intraplastidial eyespot and contained pyrenoids penetrated by thylakoid membranes (Figures 5A, B and 5D). These features are consistent with the ultrastructure of the chloroplasts in many species of phototrophic euglenids, such as *Colacium cyclopicolum *and *Euglena deses *[41,42]. However, the presence of more than one pyrenoid per chloroplast in some cells was a novel feature of *R. viridis *that has not been observed in any other species of euglenophyte so far (Figures 3A and 5A).

Nonetheless, euglenid chloroplasts arose as a consequence of an endosymbiotic relationship between a eukaryovorous euglenid and green algal prey cells that were most similar to prasinophyceans [9,20-22]. The fact that our isolate requires both photosynthesis and (*Tetraselmis*) prey cells in order to survive provides additional support for this inference. However, the relatively close relationship between the stable, euglenid-type chloroplasts and the transient *Tetraselmis *chloroplasts in *R. viridis *might create additional challenges when characterizing each of them at the genomic level. Nonetheless, the molecular phylogenetic position of *R. viridis *suggests that the genome of their stable, euglenid-type chloroplasts will offer some of the most compelling insights into the earliest evolutionary stages in the endosymbiotic acquisition of chloroplasts in euglenids and beyond.

## Conclusion

The unusual combination of features in *R. viridis *combined with its molecular phylogenetic position completely conforms to the expected transitional stage that occurred during the early evolution of phototrophic euglenids from phagotrophic ancestors. The marine mixotrophic mode of nutrition, the preference for green algal prey cells, the structure of the feeding apparatus, and the organization of the pellicle are outstanding examples of morphostasis that clarify pivotal stages in the evolutionary history of this diverse group of microbial eukaryotes.

## Formal taxonomic descriptions

Euglenozoa [43]

Euglenida [44] (ICZN)

***Rapaza *n. gen**. Yamaguchi, Yubuki & Leander 2012

### Description

Cells solitary and mixotrophic. Two heterodynamic flagella, unequal in length. Euglenoid movement with helically arranged pellicle strips. A minimum of one discoidal chloroplast surrounded by three membranes and with pyrenoids penetrated by double stacks of thylakoids. Robust extra-plastidic stigma and paraflagellar swelling. Eukaryovorous on microalgae using a feeding apparatus consisting of one rod of microtubules, a feeding pocket, and no vanes.

### Type species

*Rapaza viridis *Yamaguchi, Yubuki & Leander 2012

### Etymology

Latin "rapax", meaning seizing and grasping in reference to the feeding behavior of the cell. The adjective 'rapax' is used as substantive noun. Feminine.

***Rapaza viridis *n. sp**. Yamaguchi, Yubuki & Leander 2012

### Description

Swimming cells are slender with a tapered posterior end, 10.5-38.2 μm long (average, 19.5 μm), 2.9-15.1 μm wide (average, 8.4 μm); with two heterodynamic flagella, unequal in length, same in thickness; the longer flagellum about twice the length of the shorter flagellum; with stigma comprised of several (1 to more than 10) pigmented particles at the anterior part of the cell; ellipsoid paramylon grains free in the cytoplasm; pellicle consisting of 16 helical strips; one square-shaped whorl of exponential strip reduction; feeding apparatus comprised of a feeding rod consisting of four rows of 20 microtubules (4-6-6-4); preferred prey *Tetraselmis *sp.

### Holotype and hapantotypes

Both resin-embedded cells used for TEM and cells on gold sputter-coated SEM stubs have been deposited in the Beaty Biodiversity Research Centre (Marine Invertebrate Collection; accession number MI-PR113) at the University of British Columbia, Vancouver, Canada. All figures in the manuscript are based on the authentic culture, and Figure 1A has been selected as the holotype.

### DNA sequence

A sequence of the small subunit rRNA gene is deposited as GenBank Accession No. AB679269).

### Type locality

Pachena Beach, Bamfield, British Columbia, Canada (48° 47.551' N, 125° 06.974' W), June 18, 2010.

### Habitat

Tide pools, marine.

### Authentic culture

PRA-360. This culture is maintained in the American Type Culture Collection (ATCC), Manasas VA, USA.

### Etymology

The specific epithet, *viridis *(green), refers to the color of the stable chloroplasts and prey cells. The binomial is Latin for "green grasper".

## Abbreviations

AF: anterior flagellum; fep: feeding pocket; flp: flagellar pocket; G: Golgi body; LM: light microscope; M: mitochondrion; N: nucleus; Nu: nucleolus; Pa: paraflagellar swelling; PES: Provasoli's Enriched Seawater; PF: posterior flagellum; PR: paraxial rod; Rcp: chloroplast of *R. viridis*; Py: pyrenoid; S: starch grain; SCB: sodium cacodylate buffer; SEM: scanning electron microscope; Tcp: chloroplast of *Tetraselmis *sp.; TEM: transmission electron microscope; Ty: thylakoids in stacks.

## Authors' contributions

AY and NY collected the water samples from Pachena Beach, British Columbia; established and maintained cultures of *Rapaza virids *and *Tetraselmis *sp.; generated the LM, SEM, TEM and SSU rDNA sequence data; and drafted an initial version of the manuscript. BSL funded, facilitated, and supervised the collection, interpretation and presentation of the behavioral, ultrastructural, and molecular phylogenetic data and wrote subsequent versions of the manuscript. All authors have read, edited and approved the final manuscript.

## Supplementary Material

Additional file 1 **A movie showing euglenoid movement and flagellar motility in Rapaza viridis n. gen. et sp**. Click here for file

Additional file 2 **A movie showing Rapaza viridis n. gen. et sp. engulfing a cell of Tetraselmis sp. (1.7 MB MOV)**. This movie is three times faster than actual speed. Click here for file
